# Association between Optic Nerve Head Microcirculation and Macular Ganglion Cell Complex Thickness in Eyes with Untreated Normal Tension Glaucoma and a Hemifield Defect

**DOI:** 10.1155/2017/3608396

**Published:** 2017-03-23

**Authors:** Ayako Anraku, Kyoko Ishida, Nobuko Enomoto, Seiji Takagi, Hiroyuki Ito, Asuka Takeyama, Fumihiko Yagi, Goji Tomita

**Affiliations:** Department of Ophthalmology, Toho University Ohashi Medical Center, 2-17-6 Ohashi Meguro-ku, Tokyo 153-8515, Japan

## Abstract

*Purpose.* We evaluated the association between optic nerve head (ONH) microcirculation and macular ganglion cell complex (mGCC) thickness in patients with untreated normal tension glaucoma (NTG) and a hemifield defect. *Methods.* The medical records of 47 patients with untreated NTG were retrospectively reviewed. Laser speckle flowgraphy was used to obtain mean blur rate (MBR), a relative measure of blood flow. Average total deviation (TD), mGCC, and the circumpapillary retinal nerve fiber layer (cpRNFL) thickness were also analyzed. *Results.* All parameters corresponding to the defective hemifield were significantly lower than those corresponding to the normal hemifield. In the defective hemifield, MBR was correlated with TD, mGCC, and cpRNFL thickness. In the normal hemifield, MBR was only correlated with mGCC thickness, and multiple regression analysis showed that mGCC thickness was a significant contributing factor of the MBR. *Conclusion.* MBR was well correlated with mGCC thickness in eyes with untreated NTG and a hemifield defect. In the normal hemifield, mGCC thickness was a contributing factor of the MBR indicating that ONH circulatory dysfunction may be associated with retinal structural changes in the early stages of glaucoma. A reduction in ONH microcirculation may be an early indicator of the presence and progression of glaucoma.

## 1. Introduction

Glaucoma is considered as a multifactorial disease resulting from a combination of intraocular pressure- (IOP-) dependent and IOP-independent risk factors such as impaired blood flow, oxidative stress, and genetic background [1–3]. Previous studies have reported reduced ocular blood flow in patients with glaucoma, associated with the deterioration of the visual field [4–7]. It has also been reported that impaired blood flow is more pronounced in normal tension glaucoma (NTG) than in high tension glaucoma [8–10]. A recent report revealed that optic nerve head (ONH) microcirculation was associated with biomarkers of oxidative stress in patients with NTG [11]. While the pathogenesis of NTG remains unclear, blood flow disturbance appears to play a role in causing glaucomatous optic neuropathy (GON) [3, 12, 13].

Laser speckle flowgraphy (LSFG) utilizes the laser speckle phenomenon to noninvasively measure blood flow in ONH microcirculation [14]. LSFG provides the mean blur rate (MBR), a relative measure of blood flow. While it is not an exact measure, the MBR is proportional to blood velocity and has been used to measure relative differences in ONH blood flow [15, 16]. The MBR of the ONH has been shown to be highly correlated with absolute blood flow values, measured by the microsphere method or the hydrogen gas clearance method, in primates and rabbits [17, 18]. Measurements of ONH microcirculation in glaucoma patients using the LSFG technique have been shown to be reproducible [19]. Previous reports have shown that ONH microcirculations measured by LSFG were correlated with the mean deviation (MD) of the visual field measurements (Humphrey Field Analyzer (HFA)) [20–22] and optical coherence tomography (OCT) measurements, such as circumpapillary retinal nerve fiber layer (cpRNFL) thickness [15, 16, 21, 22] and macular ganglion cell complex (mGCC) thickness in eyes with glaucoma [23].

The thickness of the mGCC, comprising the inner three retinal layers (nerve fiber layer, ganglion cell layer, and inner plexiform layer), and the thickness of the cpRNFL are both diagnostic indicators of glaucoma [24–27]. Since a substantial decrease in the retinal ganglion cell population can occur before detectable visual field defects [28–31], mGCC thickness is expected to be an early indicator of the presence and progression of glaucoma.

We previously reported that baseline mGCC thickness was significantly thinner in eyes with rapidly progressing glaucoma than in eyes with slowly progressing glaucoma [32]. We also found that mGCC thickness of the normal hemifield in glaucomatous eyes with hemifield defects was significantly thinner than that in normal eyes [33]. A previous study using Doppler spectral-domain OCT revealed that both mGCC thickness and retinal blood flow were reduced in the normal hemifield of glaucomatous eyes with hemifield defects [34]. Another study reported that laser Doppler flowmetry detected diminished optic nerve blood flow in glaucoma suspects who did not have any manifested visual field defect [35]. These findings suggest that both structural and vascular changes occur in the preperimetric stage of glaucoma.

A previous study reported decreased blood flow in the neuroretinal rim corresponds to the regional visual field defect in NTG with hemifield defects [36]. However, to the best of our knowledge, there have been no reports on the relationship between ONH microcirculation and mGCC thickness in eyes with glaucoma and hemifield defects. Therefore, the current study was performed to investigate the association between ONH microcirculation and mGCC thickness in patients with untreated NTG and hemifield defects.

## 2. Materials and Methods

### 2.1. Study Subjects

This study was approved by the Toho University Ohashi Medical Center Institutional Review Board (number 14–60), and all study conduct adhered to the tenets of the Declaration of Helsinki. We retrospectively reviewed the medical records of patients with glaucoma who underwent LSFG ocular circulation measurements at the Department of Ophthalmology outpatient clinic at Toho University Ohashi Medical Center (Tokyo, Japan) between January 2013 and January 2016. Subjects who met all of the following inclusion criteria were included: untreated NTG with a hemifield defect, normal and open anterior chamber angles on slit-lamp biomicroscopy and gonioscopy, glaucomatous ONH appearance on stereoscopic evaluation with a corresponding visual field defect, intraocular pressure (IOP) ≤ 21mmHg throughout 1 day (measured every 3 hours) or on at least three different days, best-corrected visual acuity of at least 20/25, spherical refractive errors between −6.00 and +6.00 diopters (D), and a refractive cylindrical error within 2.00 D. Subjects who had any of the following conditions were excluded from analyses: history of intraocular surgery, intraocular eye disease (other than NTG), diabetes mellitus, systemic hypertension, or other systemic or ocular disease known to affect the visual field. If both eyes met all eligibility criteria, the eye with lower MD in the HFA test was selected.

The IOP was measured with a Goldmann applanation tonometer, and the IOP recorded on the same day as the LSFG measurements was used for the analyses. Systolic blood pressure (SBP) and diastolic blood pressure (DBP) were measured before performing LSFG measurements. The mean blood pressure (MBP) and mean ocular perfusion pressure (MOPP) were calculated as follows:

(1) $$\begin{array}{c} MBP=DBP+\frac{1}{3}\left(SBP-DBP\right),\\ MOPP=\frac{2}{3}MBP-IOP. \end{array}$$

All patients also underwent OCT measurements of both mGCC and RNFL thickness within 3 months of LSFG ocular circulation measurements.

### 2.2. Visual Field Analyses

**Figure 1:**
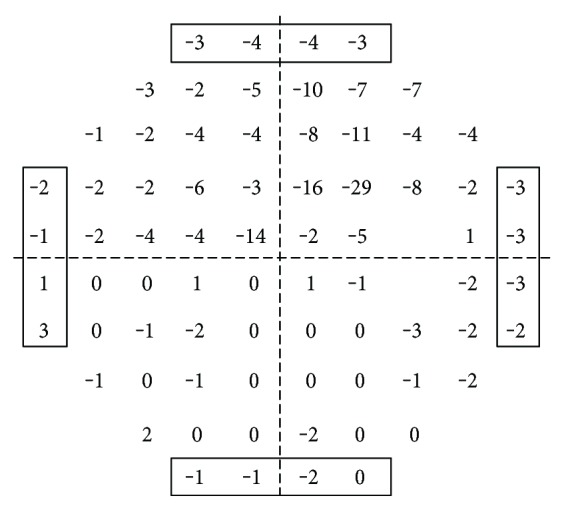
Average total deviation for the superior and inferior hemifields (29 stimuli each). The 16 edge points (boxed points) were excluded from analyses.

Standard automated perimetry was performed with an HFA (Carl Zeiss Meditec Inc., Dublin, CA, USA) using the 30-2 Swedish Interactive Threshold Algorithm. Visual field tests were considered reliable when fixation losses were <20%, false positives were <15%, and false negatives were <25%. A hemifield defect was defined as three or more significant (*P* < 0.05), nonedge, contiguous points, at least one highly significant (*P* < 0.01) point in the pattern deviation plot, and a Glaucoma Hemifield Test grading outside normal limits. A normal hemifield was defined as two or less significant (*P* < 0.05), nonedge contiguous points in the pattern deviation plot [37]. Average total deviations (TD) for the superior or inferior hemifields (29 stimuli each) were calculated. The 16 edge points were excluded from analyses (Figure 1). Average TD was used as retinal sensitivity, and dB was converted to the linear scale of 1/Lambert.

### 2.3. Optic Disc Microcirculation Measurements

**Figure 2:**
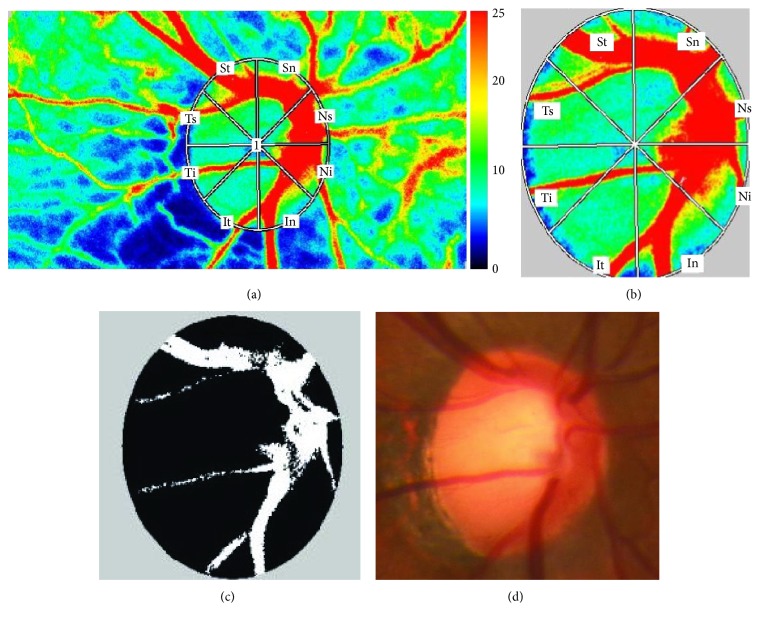
(a) Laser speckle flowgraphy false-color map. Higher numbers indicate faster blood flow. (b) Locations of the superonasal sector (Sn), superotemporal sector (St), temporal superior sector (Ts), temporal inferior sector (Ti), inferotemporal sector (It), inferonasal sector (In), nasal inferior sector (Ni), and nasal superior sector (Ns). (c) The black area represents the tissue area, and the white area represents the vessel area. (d) Fundus photograph showing the optic nerve head and major retinal vessels.

Subject pupils were dilated using 0.4% tropicamide before LSFG examination. The ocular circulation was evaluated with LSFG (LSFG-NAVI, software version 3.1.39.2, Softcare Ltd., Fukuoka, Japan), and MBR was used as a relative measure of blood flow [19]. The principle and methods of LSFG have been previously described [14, 38]. A trained operator manually determined the ONH margins with an ellipsoidal band and saved its position into the system software. The LSFG analyzer software automatically calculated the mean MBR of the three areas: mean MBR in all area of optic disc (MBR_A_), mean MBR in the vessel area of optic disc (MBR_v_), and mean MBR in the tissue area of optic disc (MBR_T_). The three values were also automatically determined for four divisions (superior, temporal, inferior, and nasal quadrants) or eight divisions: superonasal (Sn), superotemporal (St), temporal superior (Ts), temporal inferior (Ti), inferotemporal (It), inferonasal (In), nasal inferior (Ni), and nasal superior sector (Ns).  Figure 2 shows the eight divisions in a false-color LSFG map. The superior ONH circulation was an average of four sectors (Ts, St, Sn, and Ns), and the inferior ONH circulation was an average of another four sectors (Ti, It, In, and Ni).

The calculation formulas were as follows: mean MBR of the superior ONH = {(mean MBR of Ts × number of samples of Ts) + (mean MBR of St × number of samples of St) + (mean MBR of Sn × number of samples of Sn) + (mean MBR of Ns × number of samples of Ns)}/the total number of samples (Ts + St + Sn + Ns), mean MBR of the inferior ONH = {(mean MBR of Ti × number of samples of Ti) + (mean MBR of It × number of samples of It) + (mean MBR of In × number of samples of In) + (mean MBR of Ni × number of samples of Ni)}/the total number of samples (Ti + It + In + Ni).

Three consecutive measurements were taken for each subject, and the average of the measurements was used in the analyses.

### 2.4. Retinal Nerve Fiber Layer and Ganglion Cell Complex Thickness Measurements

All OCT measurements were performed with the RTVue-100 Fourier-domain OCT (software version 4.0, Optovue, Inc., Fremont, CA, USA) that uses a scanning laser diode to emit a scan beam with a wavelength of 840 ± 10 nm. This system provides images of ocular microstructures. In this study, the GCC scanning protocol was used to measure mGCC thickness. The GCC protocol consists of one horizontal and 15 vertical line scans that cover a 7 × 7 mm region. Each GCC scan captures 15,000 data points within 0.6 seconds, and a 6 × 6 mm map (corresponding to approximately 20° on the visual field map) was created. The mGCC thickness was measured from the internal limiting membrane to the outer inner plexiform layer boundary, and the OCT system provided overall superior and inferior hemifield averages.

The ONH protocol was used for cpRNFL thickness measurements. Using the fundus picture generated by OCT (a video baseline protocol), we were able to manually trace ONH contours. The RNFL thickness was automatically measured along a 3.45 mm circle centered on the center of the optic disc. A total of 775 A-scans were obtained along this circle. Our trained operator obtained good quality OCT images of each subject after pupillary dilation. Images were excluded from analyses when the signal strength was low (<40), segmentation errors occurred, or when the scan circle was not centered on the optic disc.

### 2.5. Statistical Analysis

For all analyses, the superior hemifield of the visual field corresponded to the inferior ONH circulation and the inferior hemifield of the visual field corresponded to the superior ONH circulation. Differences between the two hemifields in ONH microcirculation parameters and retinal thickness measurements (mGCC and cpRNFL) were tested for statistical significance using two-tailed, paired *t*-tests. Linear regression analyses and multiple regression analyses were used to evaluate the association between the ONH microcirculation, TD, mGCC thickness, and cpRNFL thickness. Pearson's correlation coefficients were used to evaluate the correlation between the ONH microcirculation and systemic parameters. Data were reported as mean ± standard deviation. Statistical significance was defined as *P* < 0.05.

## 3. Results

A total of 47 subjects (13 males, 34 females) with untreated NTG were included in the study. The average age was 54.6 ± 11.4 years.  Table 1 summarizes the demographic and ocular characteristics of the study subjects. Twenty-six of 47 subjects (55%) had a superior hemifield defect, and 21 of 47 subjects (45%) had an inferior hemifield defect.  Table 2 shows the average values of TD, mGCC thickness, cpRNFL thickness, and MBR corresponding to the defective and normal hemifields. All parameters corresponding to the defective hemifield were significantly lower than those corresponding to the normal hemifield.

**Figure 3:**
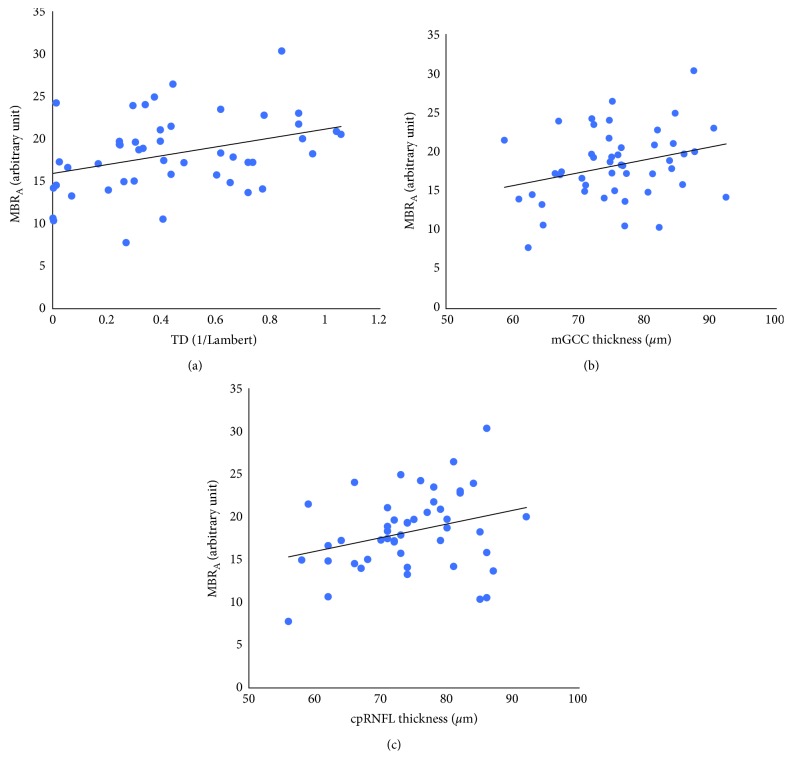
Relationships between mean blur rate in all areas (MBR_A_), total deviation (TD), macular ganglion cell complex (mGCC) thickness, and circumpapillary retinal nerve fiber layer (cpRNFL) thickness corresponding to the defective hemifield: (a) MBR_A_ and TD, *y* = 15.984 + 5.232*x* (*r* = 0.352, *P* = 0.015); (b) MBR_A_ and mGCC thickness, *y* = 5.796 + 0.165*x* (*r* = 0.293, *P* = 0.046); (c) MBR_A_ and cpRNFL thickness *y* = 6.347 + 0.160*x* (*r* = 0.299, *P* = 0.041).

Figure 3 shows the relationships between MBR_A_, TD, mGCC thickness, and cpRNFL thickness corresponding to the defective hemifield. In the defective hemifield, MBR_A_ was correlated with TD (*r* = 0.352, *P* = 0.015), mGCC thickness (*r* = 0.293, *P* = 0.046), and cpRNFL thickness (*r* = 0.299, *P* = 0.041). While MBRv was also correlated with TD (*r* = 0.302, *P* = 0.039), MBR_T_ was not (*r* = 0.212, *P* = 0.152). Regarding OCT measurements, TD was correlated with both mGCC thickness (*r* = 0.363, *P* = 0.012) and cpRNFL thickness (*r* = 0.383, *P* = 0.008). The multiple regression analysis, wherein MBR_A_ was used as the dependent variable and TD, mGCC thickness, and cpRNFL thickness were used as explanatory variables, showed that TD was a significant contributing factor (slope 5.232, 95% confidence interval [CI] = 1.062–9.402, and *P* = 0.015) of the MBR_A_.

**Figure 4:**
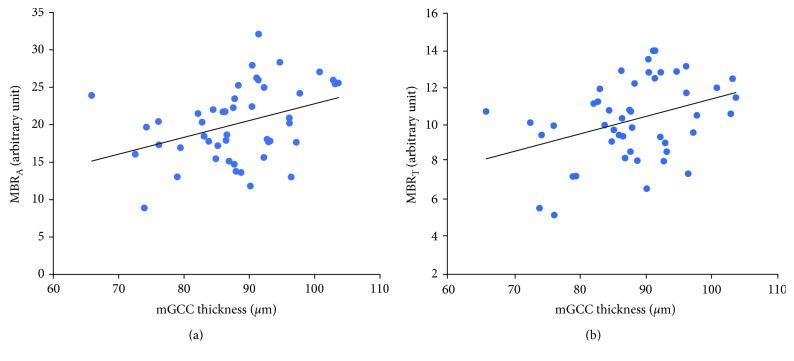
Relationships between mean blur rate in all areas (MBR_A_), mean blur rate in tissue area (MBR_T_), and macular ganglion cell complex (mGCC) thickness corresponding to the normal hemifield: (a) MBR_A_ and mGCC thickness, *y* = 0.341 + 0.225*x* (*r* = 0.371, *P* = 0.010), and (b) MBR_T_ and mGCC thickness, *y* = 1.987 + 0.094*x* (*r* = 0.361, *P* = 0.013).

Figure 4 shows the relationships between mGCC thicknesses, MBR_A_, and MBR_T_ corresponding to the normal hemifield. In the normal hemifield, only mGCC thickness was correlated with MBR_A_ (*r* = 0.371, *P* = 0.010) and MBR_T_ (*r* = 0.361, *P* = 0.013). TD was not correlated with mGCC thickness (*r* = 0.098, *P* = 0.510), cpRNFL thickness (*r* = 0.013, *P* = 0.933), MBR_A_ (*r* = 0.252, *P* = 0.088), MBRv (*r* = 0.197, *P* = 0.184), or MBR_T_ (*r* = 0.203, *P* = 0.172). The multiple regression analysis, wherein MBR_A_ was used as the dependent variable and TD, mGCC thickness, and cpRNFL thickness were used as explanatory variables, showed that mGCC thickness was a significant contributing factor (slope 0.225, 95% CI = 0.056–0.393, and *P* = 0.010) of the MBR_A_.

Table 3 shows the correlations between MBR_A_ of the entire optic disc, age, IOP, MOPP, and systemic parameters. Age was positively correlated with SBP (*r* = 0.366, *P* = 0.011) and MOPP (*r* = 0.296, *P* = 0.043), but not with MBR_A_. MBR_A_ was not correlated with IOP or MOPP.

## 4. Discussion

Previous studies have reported early structural changes in apparently normal hemifields of glaucomatous eyes with hemifield defects [33, 39, 40]. It has also been shown that a reduction in mGCC thickness corresponding to the normal hemifield is correlated with the severity of the glaucoma [41]. Sehi et al. observed reduced retinal blood flow, RNFL thinning, and mGCC loss in the perimetrically normal hemisphere of glaucomatous eyes [34]. These findings suggest that both retinal circulatory dysfunction and structural changes likely occur before retinal sensitivity reduction. Therefore, in this study, the association between ONH microcirculation and mGCC thickness was evaluated in patients with untreated NTG and a hemifield defect.

The findings revealed that MBR_A_ and MBR_T_ were correlated with mGCC thickness in the normal hemifield, although there was no correlation between MBR and TD. This indicates that ONH circulation was correlated with retinal structural changes before retinal sensitivity reduction occurred. Recently Shiga et al. reported that ONH blood flow measured by LSFG was significantly reduced in preperimetric glaucoma compared with normal subjects [22]. Another study using OCT angiography revealed that preperimetric glaucoma patients exhibited significantly lower ONH perfusion than normal patients [42]. These results suggest that impaired optic nerve blood flow develops early in the glaucomatous process. Since previous studies have reported a reduction in mGCC thickness in apparently normal hemifields of glaucomatous eyes with hemifield defects [34], we speculate that ONH circulatory dysfunction may be associated with mGCC thickness in the very early stages of glaucoma. Therefore, our results imply that detection of decreased ONH circulation and the thinning of mGCC may indicate the presence or progression of glaucoma.

In this study, MBR_A_ was correlated with TD, mGCC thickness, and cpRNFL thickness in the defective hemifield. This result is consistent with previous studies showing that ONH blood flow, as measured by LSFG, is significantly associated with the MD of HFA and cpRNFL thickness in eyes with glaucoma [15, 16, 21]. Aizawa et al. reported that MBR_A_ was an independent factor indicating glaucoma severity; however, there was no significant difference between the MBR_A_ of moderate and severe glaucomatous visual fields [21]. Chiba et al. reported that MBR_T_ was correlated more strongly with MD than MBR_V_ [15]. However, in our cohort, TD was correlated with MBR_V_, but not with MBR_T_ in the defective hemifield. While the underlying reason for this discrepancy between studies is uncertain, differences in type of glaucoma between studies may have affected the outcomes. Our study cohort comprised patients with untreated NTG, while those of Chiba et al. were treated glaucoma patients with generalized enlarged disc type.

Regarding the association between ocular blood flow and structural parameters, Hwang et al. did not find a correlation between retinal blood flow, measured by Doppler OCT, and cpRNFL thickness. However, the authors did report a paradoxical correlation between retinal blood flow and rim area measured by confocal scanning laser ophthalmoscopy [43]. They speculated that there may be a significant correlation between localized cpRNFL loss and localized retinal blood flow. However, doppler OCT used in the study assessed total retinal blood flow and it was not designed for the evaluation of the microcirculation of the neuroretinal rim. In contrast, Chen et al. reported that cpRNFL vascular microcirculation measured by OCT-based microangiography was significantly correlated with structural defects [44]. Moreover, ONH microcirculation measured by laser Doppler flowmetry was strongly correlated with ONH damage, assessed by fundus photograph and confocal scanning laser ophthalmoscopy [45]. In an animal experiment, ONH microcirculation increased during the earliest stage followed by a linear decline that was strongly correlated with loss of cpRNFL thickness [46].

The blood supply of the ONH can be divided into two main sources: the posterior ciliary artery, which feeds the prelaminar region and lamina cribrosa, and the central retinal artery, which feeds the nerve fiber layer [42]. As previously reported, the MBR_T_ is believed to correspond to the posterior ciliary artery circulation while the MBR_V_ is thought to correspond to the retinal circulation [15]. Since the penetration depth of the LSFG laser into the ONH has not been well defined, it remains speculative. However, Aizawa et al. reported that the MBR values in the ONH tissue were highly correlated with hydrogen gas clearance measurements of capillary blood flow in the ONH of albino and pigmented rabbits [18]. This suggests that the MBR_T_ can be considered an estimate of capillary blood flow in the ONH. In our cohort, mGCC thickness was correlated with MBR_T_ in the normal hemifield but not with MBR_V_. This might indicate that MBR_T_ is more affected than MBT_V_ in the early stages of glaucoma.

In the current study, both mGCC thickness and cpRNFL thickness were correlated with TD in the defective hemifield. However, there was no significant correlation between these parameters in the normal hemifield. This may be because a certain level of structural changes can occur before visual field defects are detectable. In contrast, MBR was correlated with mGCC thickness in both normal and defective fields. Therefore, ONH microcirculation might be expected to be an indicator of glaucoma both before and after retinal sensitivity reduces.

Recently, Yanagida et al. reported that the MBR of ONH was negatively correlated with age in normal populations [47]. In our cohort, age was positively correlated with SBP and MOPP, but not with MBR.

The present study has several limitations. First, the nature of this retrospective study may have introduced potential biases (e.g., our cohort had more female subjects than male). A previous study reported that the MBR of ONH in females was significantly higher than that in males in normal populations [47]. Second, due to data limitations, we were unable to identify any correlation between the MBR and central corneal thickness. Third, we divided the MBR into two sectors (superior and inferior sectors); the superior hemifield of the visual field was considered to correspond with the inferior ONH circulation, while the inferior hemifield of the visual field was considered to correspond with the superior ONH circulation. However, this may not represent actual ONH circulation. Fourth, as stated previously, the MBR remains a relative value, though it was highly correlated with absolute blood flow values measured by the microsphere method or the hydrogen gas clearance method in animal experiments [17, 18]. Furthermore, LSFG has only been employed in Japanese population to date. Finally, we only included a relatively small number of subjects in our study. Larger and prospective studies are needed in the future to confirm these findings.

## 5. Conclusions

We demonstrate that MBR is correlated with mGCC thickness in both normal and defective hemifields in eyes with untreated NTG. The multiple regression analysis revealed that mGCC thickness was a significant contributing factor of the MBR in the normal hemifield. Since previous studies have reported a reduction in mGCC thickness in apparently normal hemifields of glaucomatous eyes with hemifield defects [34], ONH circulatory dysfunction may be associated with retinal structural changes in the very early stages of glaucoma. It is uncertain whether ONH circulatory dysfunction is a cause or consequence of GON. However, a decrease in ONH circulation may be an early indicator of the presence and progression of glaucoma, in addition to mGCC thickness.

## Figures and Tables

**Table 1:** Subject demographics and ocular characteristics.

	Mean ± SD
Age (years)	54.6 ± 11.4
Gender (male/female)	13/34
Spherical equivalent (D)	−2.7 ± 2.8
Mean deviation (dB)	−3.26 ± 3.4
Pattern standard deviation (dB)	6.27 ± 4.4
IOP (mmHg)	15.1 ± 2.4
MOPP (mmHg)	44.1 ± 9.7
SBP (mmHg)	124.0 ± 19.5
DBP (mmHg)	71.2 ± 14.0
MBP (mmHg)	88.8 ± 15.0

DBP: diastolic blood pressure; IOP: intraocular pressure; MBP: mean blood pressure; MOPP: mean ocular perfusion pressure; SBP: systolic blood pressure; SD: standard deviation.

**Table 2:** Comparisons between normal and defective hemifields for total deviations, optical coherence tomography measurements, and optic nerve head microcirculation parameters.

	Defective hemifield	Normal hemifield	*P* ^∗^
Average TD (dB)	−6.0 ± 6.8	−0.4 ± 1.6	<0.001
Average TD (1/Lambert)	0.4 ± 0.3	1.0 ± 0.3	<0.001
mGCC (*μ*m)	75.6 ± 8.0	88.1 ± 8.3	<0.001
cpRNFL (*μ*m)	74.3 ± 8.4	89.0 ± 11.7	<0.001
MBR_A_ (AU)	18.3 ± 4.5	20.1 ± 5.0	<0.001
MBR_V_ (AU)	39.8 ± 8.0	41.7 ± 9.0	0.003
MBR_T_ (AU)	9.7 ± 2.0	10.3 ± 2.2	<0.001

^∗^Statistical significance determined with two-tailed, paired *t*-tests. AU: arbitrary units; cpRNFL: circumpapillary retinal nerve fiber layer; MBR_A_: mean blur rate in all area; MBR_V_: mean blur rate in vessel area; MBR_T_: mean blur rate in tissue area; mGCC: macular ganglion cell complex; TD: total deviation.

**Table 3:** Correlations between optic nerve head microcirculation, age, intraocular pressure, mean ocular perfusion pressure, and systemic parameters.

	MBR_A_		Age	
	*r*	*P*	*r*	*P*
Age	0.202	0.173	N/A	N/A
IOP	−0.090	0.547	−0.043	0.776
MOPP	−0.103	0.489	**0.296**	**0.043**
SBP	−0.111	0.456	**0.366**	**0.011**
DBP	−0.118	0.428	0.191	0.200
MBP	−0.123	0.411	0.279	0.057

DBP: diastolic blood pressure; IOP: intraocular pressure; MBP: mean blood pressure; MBR_A_: mean blur rate in all area; MOPP: mean ocular perfusion pressure; *r*, Pearson's correlation coefficient; SBP: systolic blood pressure. Values in bold are statistically significant (*P* < 0.05).
